# Invasion by *P. falciparum* Merozoites Suggests a Hierarchy of Molecular Interactions

**DOI:** 10.1371/journal.ppat.0010037

**Published:** 2005-12-16

**Authors:** Jake Baum, Alexander G Maier, Robert T Good, Ken M Simpson, Alan F Cowman

**Affiliations:** Division of Infection and Immunity, The Walter and Eliza Hall Institute of Medical Research, Parkville, Victoria, Australia; Salk Institute for Biological Studies, United States of America

## Abstract

Central to the pathology of malaria disease are the repeated cycles of parasite invasion and destruction of human erythrocytes. In *Plasmodium falciparum,* the most virulent species causing malaria, erythrocyte invasion involves several specific receptor–ligand interactions that direct the pathway used to invade the host cell, with parasites varying in their dependency on these different pathways. Gene disruption of a key invasion ligand in the 3D7 parasite strain, the *P. falciparum* reticulocyte binding-like homolog 2b (PfRh2b), resulted in the parasite invading via a novel pathway. Here, we show results that suggest the molecular basis for this novel pathway is not due to a molecular switch but is instead mediated by the redeployment of machinery already present in the parent parasite but masked by the dominant role of PfRh2b. This would suggest that interactions directing invasion are organized hierarchically, where silencing of dominant invasion ligands reveal underlying alternative pathways. This provides wild parasites with the ability to adapt to immune-mediated selection or polymorphism in erythrocyte receptors and has implications for the use of invasion-related molecules in candidate vaccines.

## Synopsis

The repeated cycles of parasite invasion and destruction of human red cells is central to malaria disease. In *Plasmodium falciparum,* the most virulent species that causes malaria, invasion involves the interaction of several parasite ligands with receptors that line the red cell surface. Central to the success of the *P. falciparum* parasite is its ability not only to utilize a number of these receptors but also to vary the primary route used. Here we show that in some parasite strains when you remove their key invasion ligands, rather than activating an alternative molecular machinery to compensate, the parasite has at its disposal a secondary means of invading that was present in the parent parasite but whose role was masked by the dominant invasion route. This suggests that the interactions that direct invasion are organized hierarchically, where silencing of the dominant ligand reveals underlying alternative means to invade. Such a mechanism gives the parasite population the ability to avoid host immune-mediated selection or to adapt to variation in red cell surface receptors and still successfully infect the human host. This stresses the importance of directing a blood-stage malaria vaccine at multiple receptor–ligand interactions to prevent parasite adaptation to invade via alternative routes.

## Introduction

Unlike many other members of the phylum Apicomplexa, malaria parasites limit their infection of host cells to the restricted population of erythrocytes in the bloodstream. The invasion of erythrocytes and the subsequent cycles of growth, replication, and rupturing of infected cells are responsible for the majority of symptoms relating to malaria disease, with severe parasite infections giving rise to rapid hemolysis and metabolic acidosis [1]. This makes the blood stage of the parasite life cycle a primary target for novel interventions to prevent invasion and combat malaria disease.

**Figure 1:**
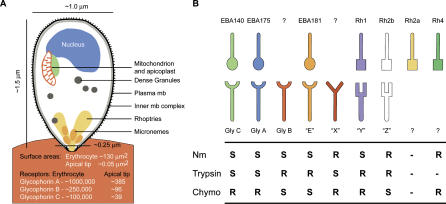
A Number of Receptor–Ligand Interactions Mediate Merozoite Invasion of the Erythrocyte (A) Estimates for the dimensions of the interaction between the apical merozoite tip and the erythrocyte surface suggest a finite number of receptors may, together with parasite ligands, direct invasion (dimensions based on [55]; receptor figures based on [56]). (B) Known receptor–ligand interactions between the invading merozoite and erythrocyte surface receptors and their enzyme sensitivities to neuraminidase (Nm), trypsin, and chymotrypsin (Chymo). S indicates sensitivity of invasion to this enzyme (inhibits invasion/ligand-binding). R indicates resistance (does not inhibit invasion/ligand-binding). Unknown ligands or receptors are indicated by a “?,” with unknown receptors E, X, Y, and Z known only from their enzyme sensitivities. Dots in table indicate that no data are available. Data are taken from [7,8,10–12,14,17,27,33,45].

Erythrocyte invasion is a rapid process governed by molecular interactions between the invading blood-stage parasite, the merozoite, and the host cell surface [2]. The merozoites bind to the erythrocyte surface, reorientate to their apical pole, and then, following formation of a tight junction between host and parasite apical tip (Figure 1A), invade, forming an isolated parasitophorous vacuole [2]. In *Plasmodium falciparum,* the most virulent of malaria species infecting humans, studies with erythrocytes modified by enzyme treatment [3–11], or from human donors lacking surface antigens [5,6,12–16], have identified a number of erythrocyte receptors used by merozoites for attachment (Figure 1B). These include glycophorins A [17], B [12], and C [14] as well as unknown receptors referred to as X [12], Y [7], Z [8], and E [10]. The dependency on these receptors for invasion is known to vary among parasites both in laboratory strains [12,15,18,19] and in isolates from the field [20–22]. Furthermore, in certain laboratory strains, such as W2mef (and its clone Dd2), the dependency on different receptors for invasion can change following either disruption of the host receptor [9] or targeted modification of the parasite ligand to which it binds [8,23].

The specialized apical complex that defines all Apicomplexan parasites contains two main vesicular bodies, termed micronemes and rhoptries (Figure 1A). Two families of parasite proteins that localize to these organelles are thought to underlie the receptor dependency of different parasite strains. The first family shares homology with the Duffy binding protein from *Plasmodium vivax* [24] and includes erythrocyte-binding antigen 175 (EBA-175) [17], EBA-140 [11,14] (also known as BAEBL [24]), and EBA-181 [10] (also known as JESEBL [24]). The EBA proteins bind sialic acid (SA) residues of glycophorins A, C, and the unknown receptor E, respectively, on the erythrocyte surface and are therefore sensitive to treatment by the sialidase neuraminidase (Figure 1B) [4,10,14,25]. The second group of proteins is a high-molecular-weight, family-sharing homology with the reticulocyte-binding proteins of *P. vivax* [26]. These include the *P. falciparum* reticulocyte binding-like homolog 1 (PfRh1) [7,27], PfRh2a, and PfRh2b [8,28], and PfRh4 proteins [29] (also known as NBP proteins −1, −2a, −2b, and −4 [7,28,29]). PfRh1 binding to the erythrocyte surface via an unknown receptor Y is SA-dependent, whereas the inferred binding characteristic of PfRh2b (to its unknown receptor Z) and PfRh4 are SA-independent (Figure 1B). Binding characteristics of PfRh2a are not known. There is also evidence for variability in the expression of the functional EBA and PfRh proteins in different parasite lines, a factor that may underlie the use of alternative invasion pathways [8,27]. Both families also include proposed pseudogenes *EBA-165* (PFD1155w) [30] and *PfRh3* (PFL2520w) [31], respectively, which are transcribed but do not appear to form protein products.

The 3D7 strain of *P. falciparum* has been fully sequenced [32], and can invade erythrocytes in both a SA-dependent and independent manner [8]. Previous studies have successfully disrupted the expression of EBA-140 [14], EBA-175 [33], PfRh1, PfRh2a, and PfRh2b [8] in 3D7 by gene knockout strategies. In each case this did not result in any measurable change in efficiency of invasion into normal erythrocytes, demonstrating that there is considerable redundancy in invasion pathways and that the role of each protein is either expendable or can be readily compensated [8,14,33]. The 3D7ΔEBA-175 parasites invade chymotrypsin-treated erythrocytes at a greatly reduced rate when compared to the parental 3D7 strain. This suggests that EBA-175 is functional in the parent, and as a result of the gene disruption, the mutant parasite has a reduced repertoire of potential invasion receptors [14,33], although on which alternative receptor(s) it is relying is not known. Disruption of *PfRh1, PfRh2a,* or *EBA-140* in 3D7 does not result in any noticeable alteration in the sensitivity of invasion, even in enzyme-treated erythrocytes, suggesting these proteins play a less important role in the parent [8,14,27]. Unlike the other knockout lines and 3D7 parent, the 3D7ΔRh2b parasite uses receptors that are chymotrypsin-resistant [8]. This suggests that an invasion pathway, not normally used by the parent strain, has been utilized in the mutant parasite. This mirrors the phenotype observed when EBA-175 function is removed in W2mef (a parasite usually sensitive to neuraminidase), resulting in a dramatic shift to SA-independent invasion [23].

Here we have analyzed the novel invasion pathway used by the 3D7ΔRh2b parasite. Our results suggest that changes in the receptor–ligand dependency of parasites are directed by a hierarchy of molecular interactions and not, at least in the case of 3D7, by the activation of new ligands. This mechanism would provide parasites with the ability to rapidly adapt to host immune selection directed at primary invasion ligands or to polymorphism in erythrocyte receptors.

## Results

### PfRh3 Is Activated in 3D7ΔRh2b Parasites

**Figure 2:**
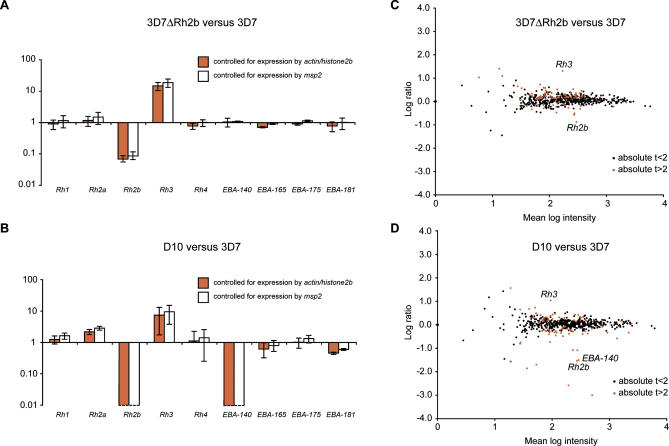
Comparison of Transcription of Key Invasion Genes in 3D7, 3D7ΔRh2b, and D10 (A and B). Expression levels of *EBA* and *PfRh* genes controlled by the relative expression of either *actin* and *histone 2b* (two constitutively expressed genes) or *msp2* (a gene expressed later on in the life cycle). Error bars represent the 95% confidence interval (CI) of values from two to three independent amplifications. (C and D) Affymetrix microarray comparison of log gene expression intensity (as measured by MOID) between cRNA isolated from late-schizont 3D7 and (C) 3D7ΔRh2b or (D) D10 parasites. Data shown are restricted to 548 genes whose expression profile matches clusters 4, 13, 14, 15 [34], including genes that encode proteins known to be involved in invasion. Color represents significance of the change in the level of gene expression as measured by the *t*-statistic (<2 black, >2 red). The control genes *PfRh2b* (knocked out) and *EBA-140* (deleted in D10) and upregulation of *PfRh3* are shown.

A number of genes that encode invasion-related proteins have been disrupted in the *P. falciparum* parasite strain 3D7 [8,14,33]. However, so far only the 3D7ΔRh2b parasite, lacking the *PfRh2b* gene, alters its invasion receptor dependency, utilizing erythrocyte receptors that are more resistant to chymotrypsin than the parent [8,11]. To investigate the molecular basis for this novel invasion pathway, transcription of key invasion-related genes in late-stage (40–48 h postinvasion) 3D7 and 3D7ΔRh2b parasites was measured. The unrelated isolate D10 was included as it naturally lacks the *PfRh2b* gene, as well as the *EBA-140* gene, and invades in a chymotrypsin-resistant manner similar to 3D7ΔRh2b [8]. The relative amount of RNA transcribed (relative to either the *actin* and *histone 2b* genes, or the late-stage expressed *msp2* gene) for the EBA *(EBA-140, EBA-165, EBA-175,* and *EBA-181)* and *PfRh (PfRh1, PfRh2a, PfRh2b, PfRh3,* and *PfRh4)* families of genes is shown in Figure 2. As predicted, transcription of the *PfRh2b* in both 3D7ΔRh2b and D10 was greatly reduced (or absent) when compared to wild-type 3D7 (Figure 2A and 2B). The absence of *EBA-140* was also confirmed for D10 (Figure 2B). Among the *PfRh* and *EBA* genes, the only gene that showed significant increases in relative levels of RNA transcript, in comparison to 3D7, was *PfRh3,* a putative pseudogene. This gene appeared to be transcribed approximately 10-fold more in both 3D7ΔRh2b and D10 relative to 3D7 (Figure 2A and 2B).

To confirm the apparent activation of *PfRh3* in 3D7ΔRh2b and D10 and investigate other genes that may have changed with the loss of the *PfRh2b* gene, we probed Affymetrix gene-chips representing 95% of the predicted genes in the 3D7 *P. falciparum* genome [34]. Global analysis of expression carried out using the MOID [35], RMA [36], and GCRMA algorithms [37] showed a number of genes that have changed their transcription levels between parasite lines (Dataset S1). Measurements from replicate chips were used to calculate a moderated *t*-statistic. The *t*-statistic is used to rank genes in order of confidence of differential expression [38]. Analysis was restricted to those genes that are expressed at similar times to genes whose products are known to be involved in invasion (clusters 4, 13, 14, and 15 [34]) but excluding the known virulence genes (*vars, rifins,* and *stevors* [39]), leaving a total of 548 genes (Datasets S1–S4). As expected, *PfRh2b* is downregulated in both 3D7ΔRh2b and D10, as is *EBA-140* in D10. To investigate which gene might have been upregulated to compensate for loss of PfRh2b, a low cutoff point of *t* greater than or equal to 2 was chosen to define those genes that have increased their transcription between the 3D7ΔRh2b or D10 parasite and 3D7 wild type. By this cutoff, and comparison between the algorithms, seven genes showed significant increase in transcriptional activity in the 3D7ΔRh2b (Figure 2C; Table 1). *PfRh3* is activated in both 3D7ΔRh2b and D10 parasites, by approximately 60-fold and approximately 15-fold, respectively (values from the MOID calculation, Figure 2D; Table 1), supporting the result observed using RT-PCR. Of the other genes that have increased in transcriptional activity (PlasmoDB ID: PFE1465w, PFB0680w, PFF0670w, PF08_0036, PF13_0173, and PF14_0567), all are uncharacterized genes with no obvious adhesive domain and lack a signal peptide and transmembrane domain. PF08_0036 encodes a putative protein transporter, which is unlikely to function directly in invasion. Additionally, and in contrast to *PfRh3,* even by the most generous estimates these genes have only increased by 4-fold in 3D7ΔRh2b and do not show up regulation in D10. Therefore, although their protein products may play an indirect role in the new invasion pathway of 3D7ΔRh2b, it is unlikely that they directly compensate for the loss of function of PfRh2b.

**Table 1:**
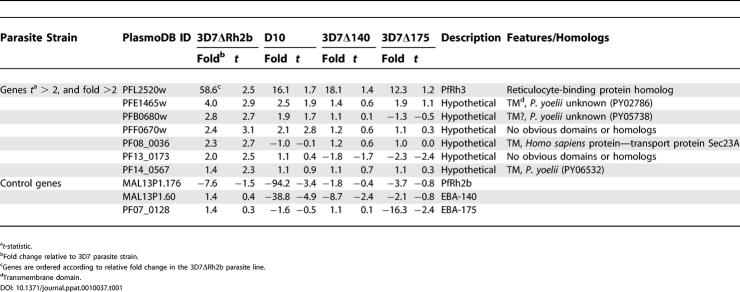
Genes That Have Altered Their Transcription Levels >2-Fold in 3D7ΔRh2b Relative to 3D7 Wild Type with a *t*-Statistic ≥2

Since *PfRh3* transcription was very low in 3D7, its elevated level in 3D7ΔRh2b and D10, both of which invade more efficiently into chymotrypsin-treated erythrocytes, may indicate a role for *Rh3* in compensating for the loss of function of Rh2b, and as such define the molecular basis for the novel chymotrypsin-insensitive pathway. However, *PfRh3* may also be activated in 3D7ΔEBA-175 and 3D7ΔEBA-40, although these parasites show no change in the phenotype or efficiency of invasion. (Table 1, Datasets S1–S5).

### PfRh3 Is Not Essential for the Novel Invasion Pathway Used by 3D7ΔRh2b

The presence of a conserved frame-shift mutation(s) in the *PfRh3* gene from multiple parasite lines [40], and the inability to detect a protein product for the gene using rabbit serum raised against recombinant portions of the gene [31] suggest that *PfRh3* is a transcribed pseudogene. To investigate whether the increase in levels of *PfRh3* transcript in 3D7ΔRh2b and D10 was associated with the production of a functional PfRh3 protein, genomic DNA and cDNA from the three strains were used as template to amplify a 5′ region of *PfRh3.* Sequences of this region, from genomic or cDNA, all retained the 5′ frame-shift mutation (data not shown), suggesting no protein product could be produced. The possibility that posttranscriptional modification might remove the frame-shift mutation from *PfRh3* mRNA, and therefore produce a functional protein product, was excluded by failure of rabbit anti-Rh3 antisera to detect any protein product from either 3D7ΔRh2b or D10 (data not shown).

**Figure 3:**
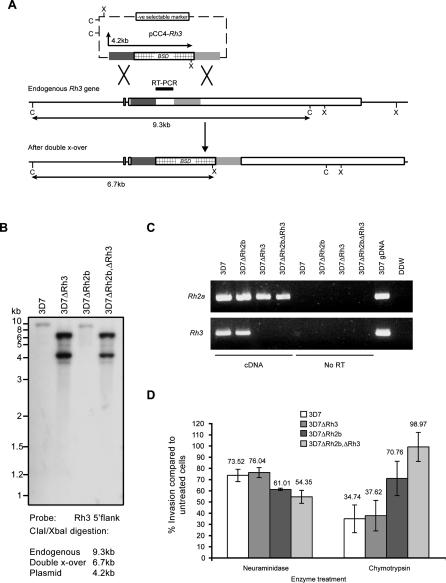
Targeted Disruption of *PfRh3* Shows It Is Not Essential for the 3D7ΔRh2b Chymotrypsin-Resistant Pathway (A) Disruption of the *PfRh3* gene in 3D7. The pCC4-*Rh3* plasmid contains the blasticidin-S deaminase selectable marker, a negative selectable marker (A. G. Maier and A. F. Cowman, unpublished data), and 5′ and 3′ *Rh3* regions. *PfRh3* is shown with homologous target sequences (shaded regions). The double-crossover integration events are shown for 3D7, resulting in the deletion of a 5′ region of the gene. Restriction enzymes are C (ClaI) and X (XbaI), with fragment sizes shown for a C/X digestion. RT-PCR amplification target is shown as a black bar. (B) Southern blot of genomic DNA from parasites shown digested with ClaI and XbaI and probed with the 5′ *Rh3* flank from the pCC4-*Rh3* vector. (C) RT-PCR of *PfRh2a* and *PfRh3* from 3D7 and three knockout lines. (D) Invasion into enzyme-treated erythrocytes expressed as a percentage of that into untreated erythrocytes for 3D7 and three knockout lines. Values above each column indicate the mean % invasion, with error bars representing the 95% CI from three independent assays.

To finally rule out the role of *PfRh3* in the 3D7ΔRh2b parasite, we constructed a plasmid (pCC4-*Rh3*) (Figure 3A) that would integrate into the *PfRh3* locus in the 3D7ΔRh2b parasite by double recombination crossover [41]. Because the original 3D7ΔRh2b knockout had been achieved using a vector containing the human *dihydrofolate reductase (hdhfr)* gene [8], the *blasticidin-S deaminase* gene (*BSD,* conferring resistance to blasticidin) was used in pCC4-*Rh3* to select for parasites carrying the plasmid. As a control, knockout of *PfRh3* was also performed in parental 3D7 since, in the absence of other modifications, this gene has been easily disrupted previously [31,41]. To confirm integration of pCC4-*Rh3* into both 3D7 and 3D7ΔRh2b, genomic DNA from parental and transfected parasites was analyzed by Southern hybridization. This revealed DNA fragments consistent with double-crossover recombination into the *PfRh3* locus (Figure 3B). This represents the first time two genes have been sequentially disrupted in *P. falciparum.* To ensure that *PfRh3* transcript had been lost from 3D7ΔRh3 and 3D7ΔRh2bΔRh3 we used RT-PCR with late-stage RNA from these parasite lines as well as 3D7 and 3D7ΔRh2b. Primers targeted to a specific region of *PfRh2a* showed consistent expression in all parasites lines (Figure 3C), while those specific for the region of *PfRh3* deleted by the gene-disruption strategy showed no expression in the *PfRh3* knockout lines, confirming that the gene has been disrupted (Figure 3C).

Finally, to confirm that *PfRh3* plays no role in the novel invasion pathway used by the 3D7ΔRh2b parasite, 3D7, 3D7ΔRh2b, 3D7ΔRh3, and 3D7ΔRh2bΔRh3 were grown in erythrocytes treated with neuraminidase and chymotrypsin. Invasion rates into untreated cells were similar for all parasite lines (data not shown). Invasion into neuraminidase-treated cells showed similar rates for the 3D7 and 3D7ΔRh3 parasites of approximately 75% to that into untreated cells and approximately 60% in the 3D7ΔRh2b and 3D7ΔRh2bΔRh3 parasites (Figure 3D). Chymotrypsin treatment similarly grouped the parasites into those in which *PfRh2b* had been disrupted with 3D7 and 3D7ΔRh3 parasites, invading at approximately 35% to that into untreated cells and between 70%–100% in the 3D7ΔRh2b and 3D7ΔRh2bΔRh3 parasites. These figures are similar to those seen in the original analyses of 3D7ΔRh2b [41] and demonstrate that *PfRh3* plays no role in the novel chymotrypsin-resistant pathway.

Therefore, despite the consistent finding of *PfRh3* up-regulation in both the 3D7ΔRh2b and D10 parasites, these data clearly demonstrate that it plays no role in determining the novel invasion pathway used by 3D7ΔRh2b. Indeed, *PfRh3* activation in both 3D7ΔEBA-140 and 3D7ΔEBA-175 (which do not appear to use this novel pathway) further argues against a role for *PfRh3* in compensating for the loss of PfRh2b function (Dataset S5). The lack of obvious candidate genes that have properties consistent with their role in invasion suggests that proteins that are already present in 3D7 mediate the previously unused pathway.

### Relative Levels of Key Invasion Proteins Have Not Changed between Wild-Type 3D7 and the 3D7ΔRh2b Parasite

**Figure 4:**
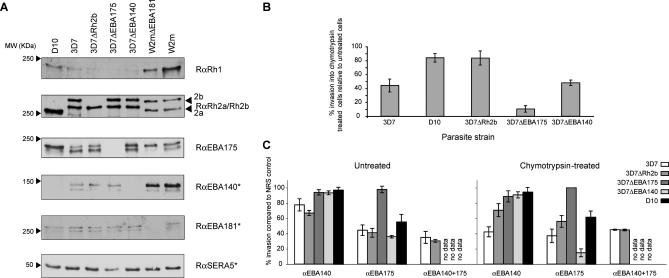
Changes in Protein Levels Do Not Underlie the 3D7ΔRh2b Chymotrypsin-Resistant Pathway (A) Western blot of parasite culture supernatant material probed with rabbit polyclonal antibodies against the functional EBA and PfRh proteins. SERA5 is used as a loading control. Antibodies marked with an asterisk (*) indicate the same gel stripped and reprobed with a different antibody. (B) Invasion into chymotrypsin-treated erythrocytes for five parasite lines represented as the percentage of invasion to that into untreated erythrocytes. (C) Invasion into either untreated or chymotrypsin-treated erythrocytes in the presence of protein-G purified polyclonal rabbit antiserum raised against recombinant EBA-140 and EBA-175 (or both together). Values are represented as the percentage of invasion in the presence of NRS. Error bars represent the 95% CI from three independent assays.

A number of parasite proteins have been implicated in mediating the alternative invasion pathways used by *P. falciparum* to invade erythrocytes [4,7,8,10,11,14,17]. Although transcript levels of these invasion-related genes do not appear to have changed with loss of the *PfRh2b* gene in 3D7, posttranscriptional modification resulting in changes in the absolute amounts of protein product may underlie the novel invasion pathway used in the 3D7ΔRh2b parasite line. Semiquantitative Western blots, where loading was controlled for expression of the SERA5 protein, were undertaken to determine if changes in absolute levels of invasion-related proteins had occurred (Figure 4A). Other than the absence of detectable protein in knockout lines (as expected), no discernable differences are present between the different 3D7 parasite lines for the PfRh1, PfRh2a, PfRh2b, EBA-140, EBA-175, or EBA-181 proteins (Figure 4A), suggesting that the absolute levels of the invasion proteins have not changed. Proteomic analysis of late schizonts from both 3D7 and 3D7ΔRh2b also failed to show any dramatic changes in the presence of any particular proteins (unpublished data). However, as with previous studies [42], invasion genes were poorly represented in the analysis, with only a few peptide fragments from one or two proteins showing up. This suggests that the absolute amount of most invasion proteins is not sufficient for detection, using current proteomic methods. This demonstrates that the absolute levels of key invasion proteins in 3D7, following the disruption of PfRh2b, appear to remain constant despite a shift in the invasion receptor dependency.

### Invasion Inhibitory Antibodies against EBA-140 and EBA-175 Inhibit 3D7ΔRh2b and the Parental 3D7 Strain

The inhibition caused by antibodies raised against recombinant EBA-140 and EBA-175 relate directly to the function of these proteins in invasion, as shown by the failure of specific antibodies to inhibit parasites where the respective genes have been disrupted [14,33]. The binding of EBA-140 and EBA-175 to the erythrocyte surface is insensitive to chymotrypsin treatment [11] since their receptors, glycophorins A and C, are resistant to digestion by this enzyme (although some glycophorin A is sensitive to chymotrypsin, as much as 60% remains following enzyme treatment because of a variable glycosylation site near the enzyme cleavage point [43]). Given that the pathway being used by 3D7ΔRh2b is relatively chymotrypsin-resistant (Figure 4B), alterations in the importance or reliance of 3D7ΔRh2b on EBA-140 or EBA-175 might underlie this unidentified pathway. To address this, invasion assays in the presence of αEBA-140 and αEBA-175 were undertaken with the 3D7 mutant parasite lines, 3D7 and D10.

As found previously [14], invasion of 3D7 into untreated erythrocytes in the presence of αEBA-140 was approximately 80% compared to that in the presence of normal (preimmunization) rabbit serum (NRS) (Table 2; Figure 4C), demonstrating a role for the EBA-140 protein despite there being no altered or observable phenotype following the disruption of its gene in 3D7 [14]. Invasion of 3D7ΔRh2b in the presence of αEBA-140 was approximately 70%, the range of which is not significantly different from that in 3D7 (Table 2; Figure 4C). Both 3D7ΔEBA-140 and D10 were unaffected by αEBA-140, confirming the specificity of the antibody [14]. Interestingly, in the presence of αEBA-140, 3D7ΔEBA-175 invasion was significantly less inhibited (approximately 90% invasion) than wild-type 3D7 or 3D7ΔRh2b (Table 2; Figure 4C). This suggests that in the absence of EBA-175, invasion by the 3D7 parasite is entirely SA-independent and no longer relies on any residual function of EBA-140.

**Table 2:**
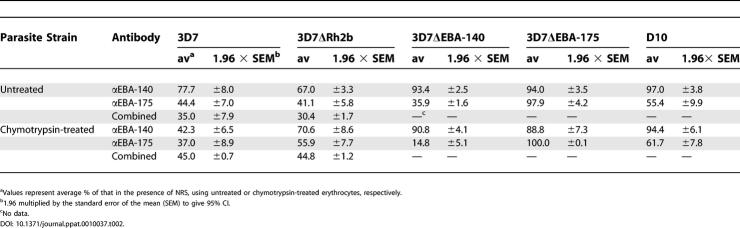
Percentage Invasion of Parasites into Untreated and Chymotrypsin-Treated Erythrocytes in the Presence of IgG-Purified Rabbit Antiserum Raised against Recombinant EBA-140 and EBA-175

The 3D7, 3D7ΔRh2b, and 3D7ΔEBA-140 parasites invaded erythrocytes at approximately 40% in the presence of αEBA-175. This adds strong support to the observation that 3D7 primarily uses the EBA-175/GYPA receptor–ligand interaction to mediate invasion despite being able to invade via alternative (SA-independent) routes when this pathway is blocked (for example following neuraminidase treatment) [33]. Inhibition of 3D7ΔEBA-140 by EBA-175 antibodies demonstrates a more dominant role for EBA-175 over EBA-140. Invasion of 3D7ΔEBA-175 was not inhibited by αEBA-175, confirming the specificity of the antibody [23].

Invasion of 3D7 into erythrocytes pretreated with chymotrypsin for all parasite lines confirmed results found previously (Figure 4B). This enzyme digests many surface receptors but does not entirely remove glycophorins C or A, the receptors of EBA-140 and EBA-175, respectively [11]. As such, by removing a number of other putative receptors, but leaving glycophorins C and A, chymotrypsin treatment will accentuate the inhibitory effects of αEBA-140 and αEBA-175. The relative inhibition of 3D7 with αEBA-140 (compared to that in the presence of NRS) was reduced to approximately 40% invasion (Table 2; Figure 4C), confirming that reported previously [14]. However, unlike 3D7, chymotrypsin treatment did not significantly reduce the relative inhibition of 3D7ΔRh2b with αEBA-140, suggesting that EBA-140 plays a less important role in the PfRh2b knockout parasites. Following chymotrypsin treatment, 3D7ΔEBA-140 and D10 showed no inhibition from αEBA-140. This was also true for 3D7ΔEBA-175, which further supports its shift to reliance on SA-independent invasion. The lack of reduction in relative invasion of 3D7ΔRh2b with αEBA-175 and chymotrypsin treatment, like that with αEBA-140, demonstrates that the PfRh2b knockout appears to be less reliant on EBA-175 than the parent strain; 3D7, the EBA-140 knockout line, and D10, in the presence of αEBA-175, all showed only a moderate to no increase in the relative inhibition compared to NRS. Finally, it is worth noting that inhibition in the presence of both antibodies together (both with and without chymotrypsin treatment) is not dramatically different from that seen in the presence of αEBA-175 alone (Table 2; Figure 4C). A caveat to the observations following chymotrypsin treatment is that our ability to detect subtle differences in invasion is limited with chymotrypsin treatment since, in both parasites, it reduces the efficiency of invasion and, as such, means that the absolute numbers of parasites are less in each assay.

Decreased inhibition of invasion in the presence of αEBA-140 and αEBA-175 demonstrates that without PfRh2b, the 3D7 parasite utilizes receptors other than glycophorins C and A. Furthermore, it supports the general assertion that in wild-type 3D7, EBA-175 plays a more critical role than EBA-140 in mediating invasion.

### Long-Term Culturing of 3D7 in Chymotrypsin and Low-Trypsin-Treated Cells Does Not Lead to a Switch in Invasion Phenotype

**Figure 5:**
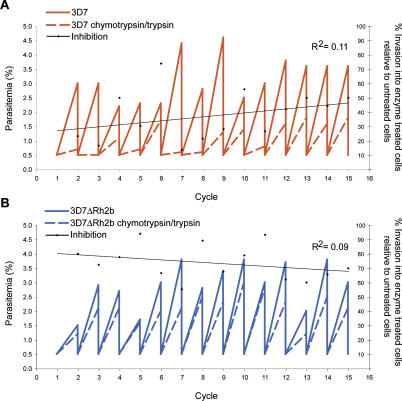
Long-Term Selection of (A) 3D7 and (B) 3D7ΔRh2b in Erythrocytes Pretreated with Chymotrypsin (1.5 mg/ml) and Trypsin (0.1 mg/ml) Shows No Significant Change in Receptor Dependency Parasitemia was measured by microscopy; then cultures were diluted down to 0.5% parasitemia at 48-h intervals. Cultures were maintained for greater than 30 d. Secondary *y*-axis represents invasion into enzyme-treated erythrocytes as a percentage of invasion into untreated erythrocytes, with a regression line plotted across the values.

Following selection on enzyme-treated cells, certain parasite lines (notably W2mef/Dd2) are able to switch their route of invasion to a previously unused pathway [9]. Having demonstrated that neither a change in transcription nor a change in protein levels, or the function (as determined by inhibition) of alternative invasion ligands, underlies the novel invasion pathway used by the 3D7ΔRh2b parasite line, long-term selection of 3D7 was undertaken to attempt to select for the chymotrypsin-resistant pathway (thereby mimicking knockout of the *PfRh2b* gene), as has been observed with selection on neuraminidase-treated cells and the knockout of EBA-175 in W2mef [23]. After selection on chymotrypsin- and trypsin-treated cells (the most marked phenotype of the new pathway in 3D7ΔRh2b) for more than 30 d, no significant change in enzyme sensitivity occurred in 3D7 to make it more like the 3D7ΔRh2b parasite (Figure 5A and 5B).

Therefore, even though the underlying mechanism for invasion into chymotrypsin- and trypsin-treated erythrocytes appears to be already present in the 3D7 parasite, it cannot be upregulated when PfRh2b is present. This suggests, that PfRh2b is the a priori ligand used by 3D7 and that the physical presence of functional PfRh2b relegates any potential role of a secondary invasion ligand(s) (and its resulting chymotrypsin-resistant pathway), reducing the invasion efficiency of 3D7 into certain enzyme-treated cells.

## Discussion

The availability of the completed *P. falciparum* genome has led to the identification of two families of proteins that are thought to underlie the variable receptor–ligand interactions used by malaria parasites to invade human erythrocytes. Gene disruption of each of these EBA and PfRh proteins have been generated in various parasite lines, and in 3D7, the genome reference malaria parasite, null mutants for *EBA-140, EBA-165, EBA-175, PfRh1, PfRh2a, PfRh2b,* and *PfRh3* are available [8,14,30,33,41]. Of these lines only the 3D7ΔRh2b parasite apparently uses an invasion pathway that is qualitatively different to that of the parent 3D7 strain [8]. Here we have used specific gene disruptions and a comparative analysis of gene and protein expression and function to demonstrate that the receptor-mediated invasion pathway of 3D7ΔRh2b likely uses ligands that are already present in wild-type 3D7, but our ability to measure their function was masked by the presence of PfRh2b.

### Compensation for the Function of PfRh2b in 3D7

Comparison of gene expression between 3D7 and 3D7ΔRh2b showed that *PfRh3,* a related *PfRh* pseudogene, does increase its mRNA expression levels significantly following *PfRh2b* disruption. However, no protein product is produced (it being a pseudogene [31]). Furthermore, the possibility that *PfRh3* might function at the RNA level is ruled out by gene disruption on a 3D7ΔRh2b genetic background. This is the first time two genes have been disrupted sequentially in the malaria parasite. Previous work analyzing *PfRh* transcription in the parasite strains FCB1, T996, and 3D7 showed that *PfRh3* levels are naturally much lower in 3D7 than the other two strains [44]. Therefore the explanation for *PfRh3*'s anomalous increase in transcription in 3D7ΔRh2b might lie with its low levels of transcription in the 3D7 parent strain, which changes in the knockout parasite. This is supported by a similar activation of *PfRh3,* albeit to a smaller extent, in the 3D7ΔEBA-140 and 3D7ΔEBA-175 parasites. The rigors of transfection and drug selection may induce changes in gene regulation across the parasite genome that nonspecifically activates the expression of *PfRh3.* This anomalous upregulation would suggest a degree of caution in the interpretation of comparative microarray data and stress the importance of following up the functionality of up- or downregulated gene products following any selection regimen.

In addition to *PfRh3,* a small number of other genes do change their transcriptional activity (although not to the same extent as *PfRh3*). However, lack of obvious domains that might have an adhesive or binding function, and the absence of a signal peptide (necessary for export to secretory organelles or the merozoite surface) or transmembrane domain would suggest that none of these genes are likely to play a direct role in invasion. This apparent absence of change also appears to be true at the protein level for the known invasion proteins. Taken together, this strongly suggests that in 3D7ΔRh2b, the chymotrypsin-resistant invasion pathway revealed in the absence of PfRh2b is the result of a redeployment of the existing ligand repertoire of the parent parasite and not any transcriptional or posttranscriptional change in other invasion ligands.

### The Role of Other EBA or Rh Proteins in the Rh2b-Independent Pathway

A plausible explanation for the 3D7ΔRh2b parasites' increased ability to invade chymotrypsin-pretreated erythrocytes is that the parasite has become more reliant on EBA-175 or EBA-140, both of which bind red cells in a chymotrypsin-insensitive manner (mediating invasion via glycophorins A or C) [11,14,23]). However, the reverse seems to be true since 3D7ΔRh2b parasites are less inhibited than wild-type 3D7 in the presence of αEBA-175 or EBA-140 antibodies (see Figure 4C). Although inhibitory antibodies against EBA-181 are not available [10], it is clear that this ligand is not involved since its unknown receptor, receptor E, is chymotrypsin-sensitive [10]. Other possible ligands that may compensate for loss of PfRh2b are the other three functional PfRh proteins. PfRh1 binds to the erythrocyte via an unknown receptor, Y, that is chymotrypsin-resistant but neuraminidase-sensitive [7,27]. However, it is present at relatively low levels in 3D7 and cannot define the entire phenotype, given that 3D7ΔRh2b invasion is only moderately neuraminidase-sensitive [8]. This leaves either PfRh2a, PfRh4 (the receptors of which are unknown or not fully characterized [45]), or another as yet uncharacterized invasion protein. Therefore, at present it is not possible to identify the invasion ligand(s) that compensate for the loss of PfRh2b.

### The Absence of the Rh2b-Independent Pathway in the Wild-Type Parent Suggests a Molecular Hierarchy Mediates Invasion

In the absence of any differences in mRNA or protein level, the inability of the wild-type parent to invade using the same pathway as the 3D7ΔRh2b parasite suggests that the presence of PfRh2b obscures other potential receptor-mediated pathways. This is supported by our inability to adapt 3D7 parasites to chymotrypsin-treated erythrocytes following long-term selection. Since 3D7 does not become more resistant to enzyme treatment, this shows that the shift toward using an alternative receptor-mediated pathway is only possible following the removal of PfRh2b but crucially not its target receptor. The lack of changes in gene expression or protein levels and inability to alter invasion in the presence of PfRh2b suggests that a molecular hierarchy operates to direct which receptor-mediated pathway is used by the 3D7 parasite to invade.

When the parasite begins to invade the erythrocyte (see Figure 1), the tight junction is likely to contain micronemal proteins such as EBA-175, EBA-181, and EBA-140 and rhoptry proteins such as the PfRhs [8,27,29], which together will direct the receptor-mediated pathway used to invade. In 3D7, EBA-175 and PfRh2b are likely to dominate these interactions, with EBA-140, EBA-181, and PfRh1 playing only minor roles as evidenced by the lack of phenotype following their functional disruption [8,14,27] (J. Thompson and A. F. Cowman, unpublished data).

For invasion by wild-type 3D7 parasites, removal of the receptors for EBA-140, EBA-175, EBA-181, and PfRh1 by neuraminidase treatment [4,7,10,11,14,27,33], results in utilization of the PfRh2b pathway. When erythrocytes are treated with chymotrypsin, the receptors for PfRh2b [8] and EBA-181 [10] are removed, but invasion proceeds via the interaction of the remaining EBA proteins and any residual PfRh1 function. It is of note that although EBA-175 and EBA-140 both function in invasion (as demonstrated by the antibody inhibition data; see Figure 4C), it is clear that EBA-175 is dominant to the role of EBA-140 [14,33], a feature that is supported by the evidence for diversifying selection operating on EBA-175 but not (detectably) on EBA-140 [46]. It is possible that the EBA proteins may compensate for and function simultaneously with Rh (as previously suggested) [47], although they are structurally distinct protein families.

For 3D7ΔEBA-175 parasites, chymotrypsin treatment dramatically reduces the efficiency of invasion in 3D7ΔEBA-175 (<10% [33]) and can be explained by the absence of the PfRh2b pathway, which is chymotrypsin-sensitive. Critically, like the parental 3D7, no invasion ligand is present that can rescue invasion following chymotrypsin treatment, suggesting PfRh2b still dominates SA-independent invasion.

Finally, for 3D7ΔRh2b parasites, pretreatment with neuraminidase removes the receptors for EBA (and PfRh1), but the chymotrypsin-resistant pathway of 3D7ΔRh2b can compensate for their absence, it being SA-independent.

The presence of a secondary receptor-mediated pathway for invasion being redeployed from a subordinate position also appears to explain the uncovering of an unrelated invasion pathway in the T994 parasite strain following disruption of the PfRh1 ligand [27]. Here, in the absence of any noticeable changes in other invasion ligands, the knockout parasite becomes more neuraminidase- and trypsin-resistant than in wild-type T994 [27]. Following the proposed model, PfRh1 function dominates invasion in T994, but with its removal (by *Rh1* disruption) a secondary ligand compensates for its lost function in the tight junction, again via a new receptor-mediated pathway.

While these data appear to explain 3D7 and T994 invasion, they clearly do not fit all the invasion phenotypes [9] associated with these ligand families. In particular, recent work with W2mef, either following selection on neuraminidase-treated cells or after knockout of EBA-175, has shown activation of another ligand PfRh4; this activation is critical for switching to the new SA-independent invasion phenotype [45].

### Functional Consequences of Utilization of the PfRh and EBA Families

Variation and expression of PfRh and EBA proteins in both laboratory and field strains is likely to underlie invasion phenotypes [8,20–22,27]. Evidence from the mouse malaria parasite, *Plasmodium yoelii,* for the differential transcription of members of the *Py235* gene family (a family closely related to the *PfRh* family) across life cycle stages [48], suggests that variable transcription may be a general feature of Rh-related proteins in *Plasmodia.* The placement of *Rh* and *EBA* genes in subtelomeric regions of the genome may allow them to be selectively switched on or off [49], as has been recently demonstrated for *Rh4* in W2mef [45]. It is therefore possible that the invasion pathways used (as determined by expression of invasion ligand) by individual clones of *P. falciparum* may be selected to change through time. This might be evidenced by changes in the invasion phenotype of infecting parasites across the lifetime of an exposed human host, akin to the situation described for virulence genes [50], arbitrated by immune-mediated selection where the most commonly used invasion ligand is constantly selected against. Thus at the population level, successful strategies for preventing malaria disease that are directed against invasion will need to include both dominant and secondary ligands to reduce the likelihood of selection for alternative pathways.

## Materials and Methods

### Parasite cultures and transfection.

*P. falciparum* asexual parasites were maintained in human erythrocytes (blood group O+) at a hematocrit of 4% with 10% Albumax II (GIBCO, San Diego, California, United States) [51]. The 3D7 was originally obtained from David Walliker at Edinburgh University. Parasites 3D7ΔRh2b, 3D7ΔEBA-175, and 3D7ΔEBA-140 were generated in previous studies [8,14,33]. D10 is cloned from Papua New Guinea isolate FC27. Cultures were synchronized as described [52].

For disruption of the *PfRh3* gene in 3D7 and 3D7ΔRh2b, parasites were transfected with 80–100 μg of plasmid (QIAGEN) as described [41]. Positive selection for transfectants was achieved using 2.5 μg/ml of blasticidin-S [53] with negative selection against the plasmid backbone using 5 fluoro-cytosine (A. G. Maier and A. F. Cowman, unpublished data).

The PCC4-*Rh3* vector for disruption of the *PfRh3* gene was constructed using pCC4, a derivative of the pHTK plasmid [41], modified to include the BSD gene [53]) under control of histidine-rich protein (hrp) 2 promoter and the *cytosine deaminase* gene (A. G. Maier and A. F. Cowman, unpublished data). The 5′ and 3′ flanks (both approximately 1 kb) for homologous recombination into the *PfRh3* gene were amplified from 3D7 genomic DNA with the primer pairs 5′-GATCccgcggGGAAGGAGTAAAGTTTCGAAGG-3′/5′-GATCtctagaCTTATCTCCCAATATTCTC-3′ (inserts a 5′ SacII site and 3′ XbaI site) and 5′-GATCgaattcGACGGATTAGTTGAAAATAAATCC-3′/5′-GATCccatggCCATCAACTAAGGTTTCATC-3′ (inserts a 5′ EcoRI site and 3′ NcoI site), respectively. The 5′ and 3′ flanks were inserted into the vector to flank the BSD cassette. Plasmid integration was confirmed by Southern blot, using a 5′ *Rh3* flank as a probe, following standard protocols.

Long-term growth assays were undertaken for cultures of 3D7 and 3D7ΔRh2b maintained in erythrocytes treated with 1.5 mg/ml chymotrypsin (Worthington Biochemical, Lakewood, New Jersey, United States) and 0.1 mg/ml trypsin (Sigma, St. Louis, Missouri, United States). Percentage parasitemia was calculated per 1,000 red cells by microscopy every 48 h at early to midtrophozoite stage. Parasitemia was adjusted to 0.5% for the next round of invasion.

### RT-PCR and microarray analysis.

Total RNA was isolated from synchronized parasites 40–48 h postinvasion, using TRIzol (Invitrogen, Carlsbad, California, United States). RNA was further purified using DNaseI digestion by passage over an RNAeasy column (Qiagen, Valencia, California, United States).

Total RNA (5 μg) was reverse transcribed either with or without SuperScript II reverse transcriptase, using random hexamers (Invitrogen). A LightCycler (Roche, Basel, Switzerland) was used to quantify cDNA using the QuantiTect SYBR Green PCR kit (Qiagen) with *PfRh* or *EBA* gene-specific primers. Serial dilutions of 3D7 genomic DNA were used as standard controls. Relative expression ratios of *EBA* and *PfRh* genes compared to three reference genes, *actin* and *histone2b* (constitutively expressed [34]) and *msp2* (transcribed late in the erythrocytic cycle [34]) were calculated for each strain. Each gene expression level was calculated as the average of three independent amplifications for 3D7 and 3D7ΔRh2b, although only two rounds were undertaken for D10. Samples with no reverse transcriptase were used to control for possible genomic DNA contamination of cDNA preparations.

RT-PCR for verification of the *PfRh3* gene deletion was as described above, except reactions were separated on 1% TBE agarose gels to determine presence or absence of cDNA product; RT-negative reactions were included to ensure amplification was from cDNA and not genomic DNA. Primers used were Rh2aRTfwd 5′-GATGAGGTCATAAAAGATAATGAG-3′, Rh2aRTrev 5′-GAACATCATCATTCGGTTCAAAAGC-3′, Rh3RTfwd 5′-CAACGAATCAAGCACGTTTACC-3′, and Rh3RTfwd 5′-CTTATCATTTCTAAGGTAGAACC-3′.

cRNA was produced from cDNA using a T7-in vitro transcription kit (MEGAscript Kit, Ambion, Austin, Texas, United States) and used to hybridize to an Affymetrix (Santa Clara, California, United States) oligonucleotide DNA microarray as previously described [34]. Signal intensity and background noise correction were measured using the MOID [35], RMA [36], or GCRMA [37] algorithms. Differential expression was assessed for each parasite strain compared to 3D7 wild type by calculating a moderated *t*-statistic for each gene using the Limma package [54]. Features of genes that changed their transcriptional activity were investigated through publicly available databases held at http://www.PlasmoDB.org and http://www.geneDB.org.

### SDS/PAGE and immunoblot analysis.

Parasite culture supernatants were obtained from cultures following schizont rupture. Proteins were separated by SDS-polyacrylamide gels and transferred to membranes (Schleicher and Schuell Bioscience, Dassel, Germany) and probed with affinity-purified rabbit polyclonal antibodies raised against PfRh1 [8], PfRh2a and PfRh2b [7], EBA-140 [11], EBA-175 [23], and EBA-181 [10]. Supernatant loading was controlled using antibodies raised against SERA5 (a generous gift from S. Miller and B. Crabb). Blots were processed by enhanced chemiluminescence (ECL; Amersham Biosciences, Little Chalfont, United Kingdom).

### Invasion inhibition assays.

Erythrocyte invasion was assayed using normal and chymotrypsin-treated cells in the presence or absence of protein G purified rabbit anti-EBA-140 (raised against the F2 domain) [14] and rabbit anti-EBA-175 (raised against the region between the F2 and the 3′ cysteine-rich domain) [23]. Ring-stage cultures were split, half of which was treated with 1.5mg/ml chymotrypsin (Worthington Biochemical), washed, and resuspended in complete medium. Invasion assays were prepared in triplicate, using 100-μl aliquots of culture at a final hematocrit of 4% and parasitemia of 0.5% in 96-well flat-bottom microtiter plates (Becton Dickinson, Franklin Lakes, New Jersey, United States). Rabbit anti-EBA-140, anti-EBA-175, or NRS was added at a final concentration of 1 mg/ml in PBS. A PBS nonserum control was included. Control wells were smeared 72 h postassay preparation to determine life cycle stage (mid- to late-trophozoites postreinvasion); 10 μl of each resuspended assay well was added to 190 μl of filtered 10 μg/ml ethidium bromide in PBS. This was mixed and incubated for 5 min at room temperature. Parasitemia was measured using a FACScan (Becton Dickinson). Assays were performed on at least three separate occasions to calculate the mean and standard error of invasion inhibition relative to the NRS control.

## Supporting Information

Dataset S1 Comparative Gene Expression Comparative gene expression levels for 3D7, 3D7ΔRh2b, 3D7ΔEBA-175, 3D7ΔEBA-140, and D10 late-stage parasites using Affymetrix gene chips. Signal intensity and background noise correction were measured using the MOID [35], RMA [36], or GCRMA [37] algorithms.
(5.1 MB XLS)

Dataset S2 Invasion Genes: MOID As in Dataset S1, but analysis is restricted to those genes that are expressed at similar times to genes whose products are known to be involved in invasion (clusters 4, 13, 14, and 15 [34]), excluding the known virulence genes (*vars, rifins,* and *stevors* [39]). Signal intensity and background noise correction were measured using the MOID [35] algorithm.
(134 KB XLS)

Dataset S3 Invasion Genes: RMA As in Dataset S2, but signal intensity and background noise correction were measured using the RMA [36] algorithm.
(134 KB XLS)

Dataset S4 Invasion Genes: GCRMA As in Dataset S2, but signal intensity and background noise correction were measured using the GCRMA [37] algorithm.
(134 KB XLS)

Dataset S5 Summary of PfRh3 Activation in 3D7ΔEBA-140 and 3D7ΔEBA-175 Parasite Lines (32 KB DOC)

### Accession Numbers

The PlasmoDB (http://plasmodb.org/PlasmoDB.shtml) accession numbers for the genes discussed in this paper are *EBA-140* (MAL13P1.60), *EBA-165* (PFD1155w), *EBA-175* (PF07_0128), *EBA-181* (PFA0125c), *PfRh1* (PFD0110w), *PfRh2a* (PF13_0198), *PfRh2b* (MAL13P1.176), *PfRh3* (PFL2520w), *PfRh4* (PFD1150c), and other genes (PFE1465w, PFB0680w, PFF0670w, PF08_0036, PF13_0173, and PF14_0567).

## Abbreviations

*BSD*: *blasticidin-S deaminase*
CI: confidence interval
EBA: erythrocyte-binding antigen
PfRh2b: *P. falciparum* reticulocyte binding-like homolog 2b
NRS: normal (preimmunization) rabbit serum
Rh: reticulocyte binding-like homolog
SA: sialic acid
