# Supplementary material for: Invasion by P. falciparum Merozoites Suggests a Hierarchy of Molecular Interactions
Source: PLoS Pathog. 2005 Dec 16;1(4):e37. doi: 10.1371/journal.ppat.0010037 (PMC1315277; doi:10.1371/journal.ppat.0010037)
Supplement: Supplementary file 1 [file ppat.0010037.sd005.doc]

**S5. PfRh3 is activated in 3D7ΔEBA-140 and 3D7ΔEBA-175 parasite lines**

Invasion of erythrocytes by 3D7 in the absence of EBA-175 (3D7ΔEBA-175 [s1]), unlike invasion by the PfRh2b knockout, is more limited than the 3D7 parental strain, invading chymotrypsin-treated erythrocytes much less efficiently. In contrast, disruption of EBA-140 (3D7ΔEBA-140 [s2]) results in no marked reduction in invasion efficiency or change in invasion phenotype. This suggests that despite both proteins having a demonstrated function in 3D7, no additional pathways have been activated to compensate for the loss of either ligand. To confirm that no invasion-related gene changed its transcriptional activity in these parasites, labeled cRNA preparations were prepared from both knockout lines and used to probe Affymetrix® gene chips. Surprisingly, *PfRh3* is also activated in both these parasites by 18- and 12-fold respectively although the *t*- statistic in each case is below the cut off point (Table 1). Additionally, a small number of unrelated genes were judged to have increased (slightly) in 3D7ΔEBA-140 and in 3D7ΔEBA-175 (two of which increase in both) (see supplemental Data S1B-D). However, none of these were deemed likely to play compensatory roles to the deleted EBA genes, lacking obvious features such as an adhesive domain, a signal peptide or a transmembrane domain that point to their having a direct role in invasion. Therefore, other than the predicted disappearance of *EBA-140* and *EBA-175* (Table 1) no obvious invasion genes appear to have altered their transcript levels significantly when compared to the 3D7 parent line in these two EBA knockout lines (Supplementary data Table S1B-D).

s1. Duraisingh MT, Maier AG, Triglia T, Cowman AF (2003) Erythrocyte-binding antigen 175 mediates invasion in Plasmodium falciparum utilizing sialic acid-dependent and -independent pathways. Proc Natl Acad Sci U S A 100: 4796-4801.

s2. Maier AG, Duraisingh MT, Reeder JC, Patel SS, Kazura JW, et al. (2003) Plasmodium falciparum erythrocyte invasion through glycophorin C and selection for Gerbich negativity in human populations. Nat Med 9: 87-92.
